# *In vitro* genomic damage induced by urban fine particulate matter on human lymphocytes

**DOI:** 10.1038/s41598-020-65785-5

**Published:** 2020-06-01

**Authors:** Alfredo Santovito, Claudio Gendusa, Piero Cervella, Deborah Traversi

**Affiliations:** 1 University of Turin, Department of Life Sciences and Systems Biology, Torino, Italy; 2 University of Turin, Department of Public Health and Pediatrics, Torino, Italy

**Keywords:** Biological techniques, Cell biology, Genetics, Environmental sciences

## Abstract

Urban air pollution represents a global problem, since everyday many mutagenic and carcinogens compounds are emitted into the atmosphere, with consequent adverse health effects on humans and biota. Specifically, particulate matter air pollution was associated with increased risks in human mortality and morbidity. In this paper, we analyse the genomic effects on human lymphocytes of different concentrations of annual Turin PM2.5 extract by an *in vitro* micronuclei assay. Samplings were collected from an urban meteorological-chemical station positioned in Turin (Italy), one of the most polluted cities in Europe. PM2.5 sampled on filters was used for organic extraction in monthly pools and successively aggregated to produce a mixture representative for a full year PM2.5 collection. Lymphocytes were exposed to four concentrations of PM2.5: 5, 10, 15 and 20 μg/mL and micronuclei, nucleoplasmic bridges and nuclear buds were scored. With respect to controls, PM2.5 significantly increased the frequencies of all analysed biomarkers at all tested concentrations, whereas the CBPI index was significantly reduced only at the concentration of 20 μg/mL. Such *in vitro* effects can both to stimulate local authorities to adopt efficient measures for air pollution mitigation and to improve human monitoring to detect early precancer lesions.

## Introduction

Epidemiological studies suggested that particulate matter (PM) air pollution in the urban environment could be associated with an increase in several diseases, including cancer^1^ and cardiopulmonary diseases, as well as with a general increased risk in human mortality and morbidity^2–4^. Indeed, in 2016, WHO reported that ischaemic heart disease account about 36% of the deaths attributable to ambient air pollution globally^5^. Furthermore, IARC classified the outdoor air pollution as carcinogenic to humans (Group 1), particulate matter was evaluated separately and was also classified as Group 1^6,7^.

PM2.5 levels, size and chemical composition varied in relation to the local main emissions and orographic conformation. The early effects of the PM2.5 human exposure are not clearly understood. It is well known that the PM adverse effects on human depends on its physical characteristics and chemical composition^8^. At cellular level, PM can induce oxidative stress as result of cell homeostasis unbalance and subsequent mitochondrial damage ^2,9,10^, whereas, at genomic level, an excessive production of reactive oxygen species (ROS) was found to be associated to DNA damage and expression, with consequent increased risk of apoptosis for the cell^11^.

PM10 and PM2.5 are two of the most discussed parameters into the air pollution evaluation^12,13^. Such particles, especially the finest fraction, carried a wide range of mutagenic, genotoxic and carcinogenic compounds. *In vitro* tests are defined indispensable first-line tools to detect the global mixture DNA-damage effect^14^. In particular, organic extracts of urban air particles were found to induce cancer in animals and mutagenic effect in bacteria, plant and mammalian on *in vitro* cells^15–18^. However, only few studies are present in literature about *in vitro* evaluation of the possible cytogenetic and genotoxic effects of fine PM on human lymphocytes^3,19^. Turin is a city located in the Po river valley, an area where air exchanges are limited by the surrounding mountains, winds are weak, and air pollutants can accumulate easily. Mainly for these reasons, Turin is one of the most polluted European cities as the other cities of the Padana Plain^20^. Despite the PM2.5 levels detected at the urban background station decreased of the 30% in the last 10 years, the decrement is not constant and the PM2.5 concentrations vary consistently during the time so they are until today serious (Table 1). Also in the last year 2017 and 2018 the average annual PM2.5 pollution in Turin was higher than limit of 20 μg/m^3^ set by the EU and obviously also of than the air guideline value of 10 µg/m^3^ suggested by WHO^21,22^.

**Table 1:** PM2.5 pollution level recorded in Turin (adapted from Città metropolitana di Torino, 2019 and ARPA, 2018)^51,52^.

Year	Mean mass (µg/m^3^)	Mean Particles number (particles/m^3^)
2014	25	9.0 × 10^9^
2015	30	9.4 × 10^9^
2015 (involved sample)	42	—
2016	24	8.1 × 10^9^
2017	27	8.7 × 10^9^
2018	22	7.9 × 10^9^
February 2018 mobile sampling station	16–68 (min-max)	—

Among the validated genotoxicity assay, the lymphocyte cytokinesis-block micronuclei (MNi) assay is included. Such test is nowadays widely used on human population exposed to environmental and occupational carcinogens^23^. Efforts were dedicated to the standard procedure definition and to control group variability evaluation^24^. Such assay could be conducted *in vitro* on human and other animal cells^24^.

Micronuclei represent smaller additional nuclei observable in the interphase cell, that form during cell division when a chromosome or a fragment of it failed to be incorporated into one of the daughter nuclei. It was observed that the natural MNi frequency varies between certain limits (ranging from 3 to 23 MNi per 1000 cells) in different populations^25^, but many shortcomings have been reported in assessment of confounding factors, such as lifestyle patterns. All these factors together with methodological variables may contribute to the large variability in MN frequencies both in exposed and in controls^24^. Interestingly, a relationship was found between high levels of MNi in peripheral blood lymphocytes and increase of cancer risk^26^.

Chromosomal instability was also measured by scoring nucleoplasmic bridges (NPBs) and nuclear buds (NBUDs). NPBs represent dicentric chromosomes or the result of a defective separation of sister chromatids at anaphase, whereas NBUDs represent the process of elimination of amplified DNA and/or excess chromosomes from aneuploid cells^27^.

Epidemiologic data are affected by various confounding factors first of all individual and population background variability.

To produce an *in vitro* evaluation under controlled exposure dose is yet necessary to simulate the genotoxic process correlated to the PM2.5 mixtures. The aim of this study was to analyse the genomic effects on human lymphocytes of different concentrations of annual Turin PM2.5 extract by an *in vitro* MNi assay.

## Materials and Methods

### PM 2.5 sampling

PM2.5 was collected, for the first 15 days, from January to December 2015 in a meteorological-chemical station located in a background urban area in southern Turin using a sampler (Analitica Strumenti, Pesaro, Italy), according to directive UNIEN14907.

Particulate was collected on glass micro-fibre filters (Type A/E, 8″ × 10″, PALL Corporation, 25 Harbor Park Drive, Port Washington, NY 11050, USA) at a flow rate of approximately 500 L/min. The samples were collected over a 24-h period, and sample duration was controlled by a timer that was accurate to ±15 min. The exact flow was calculated daily and corrected for variations in atmospheric pressure and actual differential pressure across the filter. The filters were conditioned for 48 h and weighed using an analytical balance (±10 μg) before and after sampling to calculate the air sampled. The procedures were conducted according to the European Standard^12^.

The sampling station was located in an urban area that was not directly exposed to any relevant emission source; therefore, the PM concentration was representative of the exposure level of the resident population, without accounting for specific traffic-related or industrial exposure.

The collected filters were pooled to obtain a unique half-month organic sample resulting in a quite higher PM2.5 levels than full year monitoring data (Table 1). Extractions of each pooled sample were performed using 80 cycles of a Soxhlet apparatus (BUCHI B-811, Savatec, Torino, Italy) with acetone (product number 439126 Sigma-Aldrich), followed by evaporation induced by a Rotavapor (Savatec, Torino, Italy) instrument and resuspension of the sample with dimethyl-3-sulfoxide (product number 13409023 Sigma-Aldrich) to obtain an equivalent concentration equal to 0.2 m^3^/μL. An equal volume of extracts was included in an annual mixture, to obtain and unique sample to represent an annual PM2.5 organic extract.

The genotoxicity consists in a complex system of modifications including genetic and epigenetic alterations and the human cancer risk is determined by the accumulation of the damages during the time. In this article not so elevated exposure dose *in vitro* was used. Moreover, the involved mixture represents an annual collection, so the results can produce data linked to medium long time of exposure.

### Subjects

Peripheral venous blood from 8 healthy females (mean age ± S.D., 22.75 ± 1.28) was collected. All subjects were non-smoking, not alcoholics, not under drug therapy, and with no recent history of exposure to mutagens. Informed consent was obtained from all blood donors. The study was approved by the University of Turin ethics committee and was performed in accordance with the ethical standards laid down in the 2013 Declaration of Helsinki.

### Blood sample collection and lymphocyte cultures

About 10 mL of peripheral blood per subjects, obtained by venepuncture, were collected in heparinised tubes, cooled (4 °C) and processed within 2 h after collection. Heparinised venous blood (0.3 mL) was cultured for 72 hrs, whereas cells were fixed and collected, using protocol described in a previous published article^28^.

After 24 h of incubation, 5, 10, 15 and 20 µg/mL of air extract were added to the lymphocyte culture. The choice of such concentrations was based on previous literature on *in vitro* assay^29^ and excluded high cytotoxic effects^30^. Moreover relatively PM2.5 concentration tested on the lymphocytes was used taking in account the few studies available on lung deposited surface area, a newer unit to define the outer surface of particles influencing cells in the respiratory tract. Such evaluation shows an increment during the time as the particle number in the PM2.5 and so also of the lung deposited surface area as indicator of the PM2.5 penetrability and of the potential interaction both with the alveolar tissue and systemic blood circulation^31^.

Three control cultures were assessed: (1) positive control, by adding only MMC (final concentration 0.1 µg/mL culture); (2) 0.1% DMSO solvent control, obtained by adding 8.6 µL of dimethylsulfoxide (DMSO) to the lymphocyte culture; (3) negative control culture without both PM extract and DMSO, obtained adding 8.6 µL of RPMI medium to the lymphocyte culture.

Microscope analysis was performed at 400× magnification on a light microscope (Dialux 20, Leitz, Germany), whereas the check and photos of the damage cells were performed at 1,000× magnification. MNi, nucleoplasmic bridges (NPB) and nuclear buds (NBUD) were scored in 2,000 binucleated lymphocytes with well-preserved cytoplasm per subject (total 16,000 binucleated cells per concentration). A total of 2,000 lymphocytes per donor per concentration were scored to evaluate cytokinesis-block proliferation index (CBPI), as described in Fenech^32^.

### Statistical analysis

Comparison of mean values of the percentage of cells with MNi, NPB, NBUD, and CBPI between exposure levels and their controls was assessed by the non-parametric Mann-Whitney test and regression analysis. Statistical calculations were carried out using the SPSS software package program (version 24.0, Inc., Chicago, IL, USA). All *P* values were two tailed, and *P* values of 5% or less were considered statistically significant for all tests carried out.

### Ethical approval and Informed consent

All procedures performed in studies involving human participants were in accordance with the ethical standards of the institutional and/or national research committee and with the 2013 Helsinki declaration and its later amendments or comparable ethical standards.

### Informed consent

Informed consent was obtained from all individual participants included in the study.

## Results

**Figure 1:**
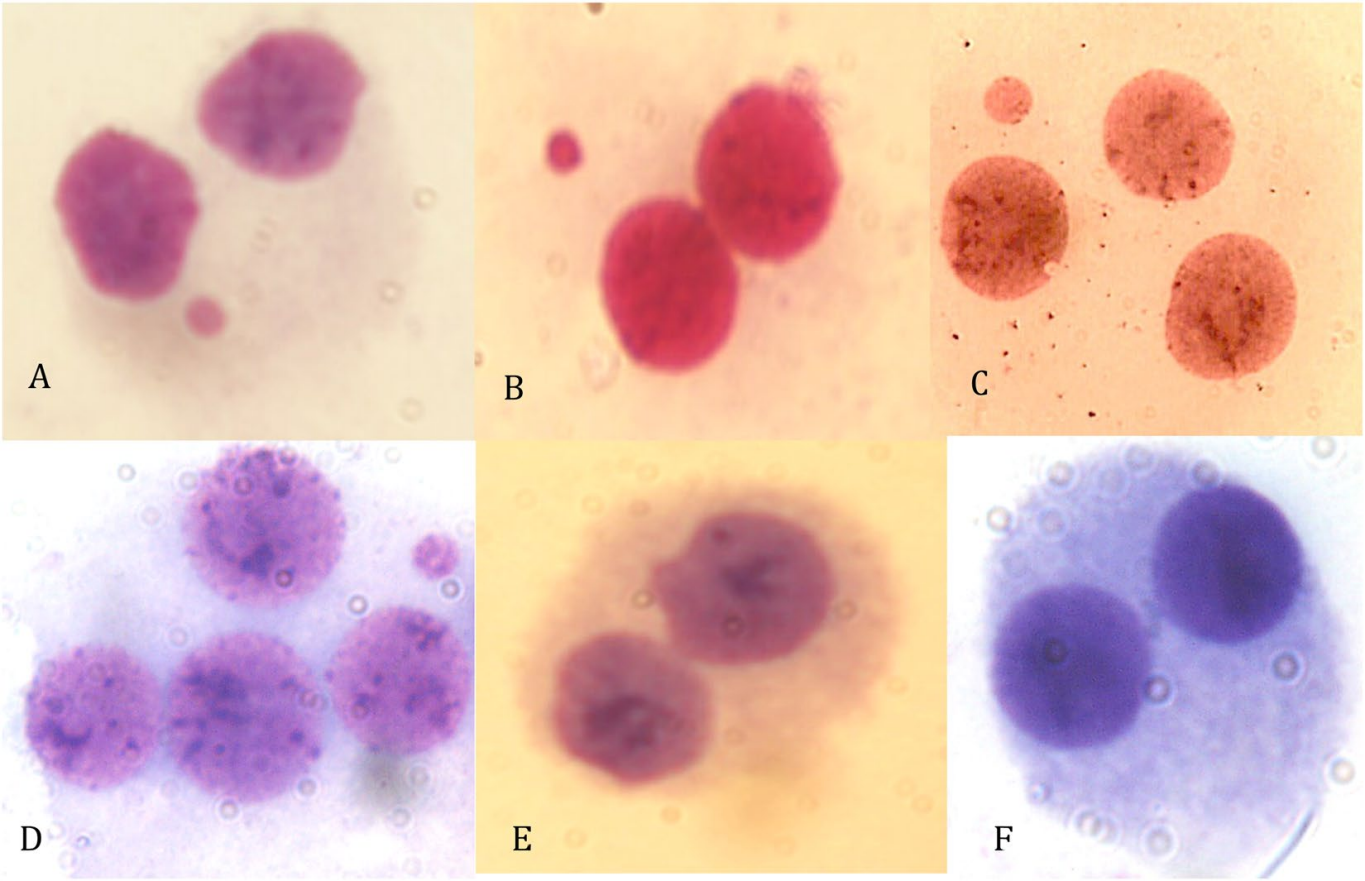
Examples of observed bi-nucleated cells with micronuclei (**A**,**B**); tri-nucleated cell with micronucleus (**C**); tetra-nucleated cell with micronucleus (**D**); bi-nucleated cell with Nuclear Bud (**E**); and bi-nucleated cell cells with Nucleoplasmic Bridges (**F**). According to standardized procedures, micronuclei of tri- and tetra-nucleated cells were not scored in the evaluation of the total genomic damage. Photos were performed at 1000X magnification.

Table 2 shows values of MNi found in the human peripheral lymphocytes cultured when exposed to different concentrations of PM2.5. In Fig. 1 examples of observed bi-, tri- and tetra-nucleated cells with MNi, as well as of NPB and NBUD were reported.

**Table 2:** Induction of micronuclei by PM2.5 in human lymphocytes *in vitro*. N = 8.

Test substance	Treatment Period Dose (h) (μg/ml)		BNCs scored	Distribution of BNCs according to the number of MNi				MNi	Ab.C	MNi/cell ± S.D. (%)	Ab.C/cell ± S.D. (%)	CBPI ± S.D.
				1	2	3	4					
NC	—	—	16,000	21	0	0	0	21	21	0.131 ± 0.026	0.131 ± 0.026	1.704 ± 0.053
0.1% DMSO	48	—	16,000	30	0	0	0	30	30	0.188 ± 0.058	0.188 ± 0.058	1.576 ± 0.185
MMC	48	0.10	16,000	149	22	6	0	211	203	1.319 ± 0.324	1.269 ± 0.214	1.327 ± 0.049 ***
PM2.5	48	5	16,000	64	7	0	0	78	77	0.488 ± 0.203*	0.481 ± 0.193*	1.504 ± 0.156
PM2.5	48	10	16,000	106	8	2	0	128	125	0.800 ± 0.220**	0.781 ± 0.194**	1.426 ± 0.126
PM2.5	48	15	16,000	142	9	3	0	169	163	1.056 ± 0.387**	1.019 ± 0.353**	1.439 ± 0.041
PM2.5	48	20	16,000	137	26	9	0	216	209	1.350 ± 0.387**	1.306 ± 0.357**	1.337 ± 0.078***

BNCs = Binucleated cells; MNi = micronuclei; Ab.C = Aberrant cells (cells with 1 or more MNi); NC = Negative Control;

MMC = Mitomycin-C; S.D. = Standard Deviation. CBPI = Cytokinesis-Block Proliferation Index.

*P = 0.003; **P < 0.001; ***P = 0.036 with respect to DMSO solvent control.

Observed MNi frequencies (±SD) were 0.488 ± 0.203, 0.800 ± 0.220, 1.056 ± 0.387 and 1.350 ± 0.387 for 5, 10, 15 and 20 μg/ml, respectively, whereas for negative control, DMSO and MMC we found frequency values of 0.131 ± 0.026, 0.188 ± 0.058 and 1.319 ± 0.324, respectively.

Similarly, the frequencies (±SD) of aberrant cells resulted 0.481 ± 0.193, 0.781 ± 0.194, 1.019 ± 0.353, 1.306 ± 0.357 for 5, 10, 15 and 20 μg/ml, respectively, and 0.131 ± 0.026, 0.188 ± 0.058 and 1.269 ± 0.214 for negative control, DMSO and MMC, respectively.

The observed values of CBPI were 1.504 ± 0.155, 1.426 ± 0.117, 1.439 ± 0.039 and 1.337 ± 0.021 for 5, 10, 15 and 20 μg/ml, respectively, and 1.704 ± 0.082, 1.576 ± 0.155 and 1.327 ± 0.026 for negative control, DMSO and MMC, respectively.

Frequency values (±SD) of cells with NPBs were 0.750 ± 0.463, 1.125 ± 0.231, 1.188 ± 0.259, 1.250 ± 0.225 for 5, 10, 15 and 20 μg/ml, respectively, and 0.313 ± 0.259, 0.500 ± 0.267, 2.188 ± 0.753 for negative control, DMSO and MMC, respectively.

Finally, for cells with NBUDs we found frequency values of 0.875 ± 0.518, 1.250 ± 0.378, 1.313 ± 0.259 and 1.833 ± 0.516 for 5, 10, 15 and 20 μg/ml, respectively and 0.563 ± 0.320, 0.813 ± 0.372 and 2.750 ± 1.165 for negative control, DMSO and MMC, respectively.

Our results indicated that PM2.5 significantly increased (*P* < 0.01) the MNi, and Ab.C formation at all tested PM2.5 concentrations (Table 2). A similar trend was also observed for NPBs and NBUDs that resulted significantly increased at all concentrations of PM2.5, with exception for the lowest concentration of 5 μg/mL (Table 3). For all these markers, a dose-effect was observed, as evidenced by the regression analysis (Table 4).

**Table 3:** Induction of NPBs and NBUDs by PM2.5 in human lymphocytes *in vitro*. N = 8.

Test substance	Treatment Period Dose (h)		BNCs scored	BNCs with NPBs ± S.D. (‰)	BNCs with NBUDs ± S.D. (‰)
NC	—	—	16,000	0.313 ± 0.259	0.563 ± 0.320
0.1% DMSO	48	—	16,000	0.500 ± 0.267	0.813 ± 0.372
MMC	48	0.10 μg/mL	16,000	2.188 ± 0.753	2.750 ± 1.165
PM2.5	48	5 μg/mL	16,000	0.750 ± 0.463	0.875 ± 0.518
PM2.5	48	10 μg/mL	16,000	1.125 ± 0.231*	1.250 ± 0.378**
PM2.5	48	15 μg/mL	16,000	1.188 ± 0.259*	1.313 ± 0.259 ***
PM2.5	48	20 μg/mL	16,000	1.250 ± 0.225*	1.833 ± 0.516 *

BNCs = Binucleated cells; NPBs = Nucleoplasmic Bridges; NBUDs = Nuclear Buds;

NC = Negative Control; MMC = Mitomycin-C; S.D. = Standard Deviation.

*P < 0.001; **P = 0.049; ***P = 0.021.

**Table 4:** Multiple regression analysis between PM2.5 concentrations.

Biomarker	β-co	95% CI (Lower) – (Upper)	*P*-value
MNi	0.736	(3.739)–(7.636)	<0.001
Cells with MNi	0.749	(3.638)–(7.212)	<0.001
NPBs	0.440	(0.074)–(0.551)	0.012
NBUDs	0.628	(0.310)–(0.840)	<0.001

MNi = Micronuclei; NPBs = nucleoplasmic bridges; NBUDs = nuclear buds.

As expected, MMC was found to significantly (p < 0.01) increase the frequency of MNi, Ab.C and NBUDs with respect to 5, 10 and 15 µg/mL of PM2.5, as well as with respect to DMSO and negative control, whereas at 20 µg/ml of PM2.5 no significant differences were observed (Tables 2 and 3). Indeed, at this last concentration of PM2.5 MMC showed significant difference only in terms of NPBs frequency (Table 3).

Finally, the DMSO solvent-control cultures did not show any difference with respect to negative controls in the frequency of all evaluated markers (Tables 2 and 3), confirming that at this low concentration DMSO has no cytogenetic effects evaluable by MNi test.

## Discussion

Air pollution represents a global problem, especially in urban areas. Everyday a lot of xenobiotics with mutagenic and carcinogens properties are emitted into the atmosphere and most of them were found to induce adverse health effects on humans and indigenous biota^33,34^.

PM could represent a “shuttle” for several compounds, such as heavy metals and hydrocarbons but also for biomolecules and pathogens. Human exposure was associated with increased levels of oxidative stress^2,3,35^, cardiovascular and infective diseases, as well as of cancer, in particular, related to PM pollution, lung cancer^35–38^.

PM2.5 is also strictly correlated to lung deposition area especially into urban environment highlighting the ability of such mixture to interact with the alveolar surface area reaching the blood circulation^39^. The high levels of PM recorded in Turin and in general in the north Italy forced to produce strictly regulation for the emission reduction but also forced to an accurate early evaluation of the PM2.5 mixture toxicity^22,40^, including genotoxicity. Such evaluation *in vitro* and with validated methods could be a proxy of the sanitary effect and human health impact for the exposed population. Early effects are not so clear in human biomonitoring study also for the complexity of the elaboration models that includes a wide range of confounding factors, including background levels.

Results obtained in the present study evidenced a possible genotoxic effect of fine PM2.5 on human lymphocytes, at all tested concentrations. Showed data seem to be concordant with results obtained by other authors although these last used different assays (chromosomal aberration test or Comet assay) and observed a significant increase of genotoxicity at generally higher concentrations of PM (for example 33, 100, 300 µg/mL)^14,41,42^. The mechanism by which PM induces genotoxicity, could be linked to the production of ROS. Indeed, it is known that inhalation of PM, particularly PM2.5 and smaller, leads to inflammation and subsequent increase of ROS production that, in last analysis, lead to base changes, mutations and/or DNA breaks^43,44^. The attack of ROS to the DNA strands can lead to double-strand DNA breaks and consequently to the formation of micronuclei. Another indirect mechanism of induction of DNA double-strand breaks is associated with formation of DNA adducts. Adducts may cause persistent blockage of one DNA strand during its synthesis and uncoupling of the other strand, which may result in the formation of double-strand breaks^44,45^.

In recent years, many genotoxicity studies focused their attention not only on the evaluation of the MNi frequencies, but also on new end-points such as NPBs and NBUDs (Fig. 1). In our study we found that PM2.5 significantly increased the NPBs and NBUDs formation at all tested concentrations, with exception of 5 μg/mL. These data assume a particular importance if we consider the fact that both NPBs and NBUDs, like MNi, are indices of genomic instability and were found to be associated with increased risk of cancer^46^.

Finally, the CBPI showed significant difference only at the highest concentration of 20 μg/mL, whereas at the other tested concentrations, PM does not seem to produce effects on the proliferation index. This result differs from data obtained by Wei and Meng^19^, although they observed a significant decrease of the Mitotic Index at concentrations higher than those we tested. This difference can be explained by a different composition of the mixture, highlighting that nor the particle quantity either the particle size is sufficient to describe toxic properties of such environmental mixture. On the other hand, a total chemical characterization is not possible in a monitoring view, for the high number of different aero dispersed toxic compounds and the chemical analysis cost.

The present study includes some limitations in study design.

First of all, the *in vitro* model can’t be perfectly overlapping an *in vivo* effect. *In vitro* study does not claim to directly describe the effects of air pollution on exposed humans. There is a wide range of physiologic protection and of repair and homeostatic mechanisms involving in human health. Similarly, also the employment of *in vitro* test can be useful to integrate data of the routine air monitoring, to describe the potential impact of pollution mixtures. On the other hand, ecological investigation recently showed higher background levels of MNi^47^. Further biomolecular epidemiologic studies, including an accurate individual exposure measure and genotoxic biomarker assessment, are auspicial.

Secondly, the samples are limited using a medium-flow sampler and only organic extraction was included. In the literature, the organic extraction showed a higher genotoxic compounds extraction efficiency^15,16,48,49^. However, when there is a specific purpose, the double extraction can be preferred, for example for natural source metals. Moreover, other work performed by colleagues in the same urban environment, showed a relevant role of water extract for oxidative stress induction but not for the DNA damage^42^. Finally in our opinion early genotoxic effect evaluation could produce a valid choice able to estimate the global genotoxicity observable as results of long time of exposure.

## Conclusions

The results herein reported showed cytogenetic effects of PM2.5 on cultured human lymphocytes in terms of increased MNi, NPBs and NBUDs frequencies. Despite the limitations of an *in vitro* study, results obtained in the present paper are interesting considering both the number of people exposed in a city as Turin with a high demographic density (331 inhabitants/Km^2^) and the additional amount of exposure due to PM2.5 pollution respect the European reference level (meanly in the last 5 years +30%). Such problem is well knowing by the regional authority and an additional plan for the reduction of air pollution was just approved during the first months of 2019. Air pollution and climate change is one of the 10 priorities for global health highlighted by WHO for the 2019^50^. Indeed, the ongoing climate changes have produced a reduction in rainfall levels throughout northern Italy and an average increase in temperatures which, associated with high levels of vehicular traffic and the use of domestic heating in winter, produced a significant increase in the concentrations of pollutants and in particular of fine PM. In this scenario, the results of this study could be a further stimulus for the adoption of more stringent measures able to reduce the presence of PM in the environment and to minimize its adverse effects on human and ecosystem health.
